# Supplementary material for: A Preferential Attachment Paradox: How Preferential Attachment Combines with Growth to Produce Networks with Log-normal In-degree Distributions
Source: Sci Rep. 2018 Feb 12;8:2811. doi: 10.1038/s41598-018-21133-2 (PMC5809396; doi:10.1038/s41598-018-21133-2)
Supplement: Supplementary file 1 — Supplementary Information [file 41598_2018_21133_MOESM1_ESM.pdf]

Supplementary Information File for A Preferential Attachment Paradox: How Preferential Attachment Combines with Growth to Produce Networks with Log-normal In-degree Distributions

Paul Sheridan[1,*] **and Taku Onodera**[2]

[1]Hirosaki University, Department of Social Medicine, Hirosaki, 036-8562, Japan
[2]The University of Tokyo, Human Genome Center, Tokyo, 108-8639, Japan
[*]paul.sheridan.stats@gmail.com

Supplementary Note 1

To understand why the form of $P_{\text{APS}}(k)$ is inconsistent with a power-law, consider on the one hand that the log-transformed version of $\mathscr{L}(k;\beta_0,\beta_1,\beta_2) = \beta_0 \exp\left[-\beta_1 \log(k) - \beta_2 \log^2(k)\right]$, i.e., $\log \mathscr{L}(k;\text{—}) = \log(\beta_0) - \beta_1 \log(k) - \beta_2 \log^2(k)$, is easily seen to have negative curvature for all $k \in \mathbb{R}^{>1}$ so long as $\beta_1, \beta_2 > 0$. Thus the curvature, i.e., the second derivative, of the log-normal form fitted by Redner is negative. On the other hand, let us consider the curvature of the cumulative of a log-transformed power-law. First note that the cumulative of a power-law distribution with scaling exponent γ is again a power-law with scaling exponent $\gamma + 1$[1]. Second, on a double logarithmic scale a power-law follows a straight line with slope equal to the negation of the scaling exponent. That a power-law cumulative has zero curvature on a double logarithmic scale follows trivially from the fact that the curvature of a straight line is zero. The conclusion that the APS citation distribution does not follow a power-law is inescapable.

Supplementary Note 2

There are a couple of technical points to be noted about the plot of Fig. 2. First, the attachment rate slopes therein differ noticeably from those found in Redner's paper. The slopes in our plot range from about 0.05 to 0.15 in the region when k is less than 150, while the corresponding range is roughly 0.10 to 0.25 for the slopes in Redner's plot. This discrepancy, peculiar as it may be, has no bearing on the conclusion that preferential attachment is present in the APS citation network. Most likely it is due to Redner having employed a variation of Eq. (2) when measuring the attachment rates. Second, we obtained substantially more noise in the large k regions, than did Redner. The discrepancy may be indicative of a failure on our part to replicate his smoothing filter in every detail, although this is unclear. In any case, it likewise has no bearing on the conclusion.

Supplementary Note 3

We provide a proof of the claim that Redner's model, as described in the main text, generates networks with in-degree distributions that asymptotically follow the log-normal distribution. The proof was communicated to the authors by Sidney Redner in an email correspondence. It is reproduced below in a form that is appropriate to our modelling framework.

A Redner's model network is generated by means of the incremental addition of nodes and edges to the singleton graph over a fixed number of discrete time-steps, $t = 1, 2, \ldots, T$. The full generative process is conveniently cast in terms of a stochastic sequence of networks, which we denote by $\{G_t\}_{t=1}^{T}$, that culminates in a T-node instantiation of the model. The said sequence is initialised with the singleton graph at time-step $t = 1$. Any subsequent transition from G_t to G_{t+1} is realised by augmenting G_t with a new node that connects with $m_t \leq t$ different nodes already present in the network to form G_{t+1}. The mean value m of the m_t's is constant over time with finite variance as t becomes large. The m_t nodes to which the new node connects are sampled from G_t without replacement with probability according to the nonlinear attachment function defined by Eq. (7). We show that the in-degree distribution $P(k)$ of networks generated in this manner are bound to follow the log-normal distribution in the tail region of k in the limit of large T.

The proof follows closely the so-called master equation approach expounded in Krapivsky and Redner[2]. The evolution of the in-degree distribution $P_t(k) \propto n_t(k)$ in Redner's model is described by the master equation

$$\frac{dn_t(k)}{dt} = \frac{n_t(k-1)A(k-1) - n_t(k)A(k)}{Z_t} + \delta_{k,m_t} \tag{1}$$

where $n_t(k)$ is the expected number of in-degree k nodes at time-step t, $A(k)$ is the attachment function of Eq. (7), $\delta_{i,j}$ is the Kronecker delta function, and $Z_t = \Sigma_{i\geq 0} n_t(i)A(i)$ is a constant of normalisation. Krapivsky, Redner, and Leyvraz argue in a related work that $n_t(k)/t$ converges to $n(k)$ as $t \to \infty$ and Z_t grows linearly in t as $Z_t = \lambda t$, so long as the attachment function does not increase faster than linearly in k[3]. In particular, this holds for the attachment function of Eq. (7). Substituting $n_t(k) = tn(k)$ and $Z_t = \lambda t$ into Eq. (1) yields

$$n(k) = \frac{n(k-1)A(k-1) + \lambda \delta_{k,m}}{\lambda + A(k)} \tag{2}$$

for $k \geq 0$ with the conventions that $A(-1) = 0$ and $n(-1) = 0$. We should also note that m_t has been replaced by its expected value m. Krapivsky and Redner solve Eq. (2) for $n(k)$ to obtain

$$n(k) = \frac{\lambda}{A(k)} \prod_{i=m}^{k} \left(1 + \frac{\lambda}{A(i)}\right)^{-1}. \tag{3}$$

To be specific, it is the special case of Eq. (3) when $m_t = 1$ for all t that they actually derived[2].

Now let us show that Eq. (3) simplifies to a log-normal distribution when k is large. By substituting Eq. (7), i.e., $A(k) = (k+1)/(1+\beta \log(k+1))$, into Eq. (3), writing the product as the exponential of a sum, converting the sum to an integral, and invoking an asymptotic approximation in the evaluation of the integral, we obtain:

$$\begin{aligned}
n(k) &\sim \frac{\lambda}{k+1} \prod_{i=m}^{k} \left(1 + \frac{\lambda(1+\beta \log(i+1))}{i+1}\right)^{-1} \\
&= \frac{\lambda}{k+1} \exp\left[-\sum_{i=m}^{k} \log\left(1 + \frac{\lambda(1+\beta \log(i+1))}{i+1}\right)\right] \\
&\approx \frac{\lambda}{k+1} \exp\left[-\int_{m}^{k} \log\left(1 + \frac{\lambda(1+\beta \log(i+1))}{i+1}\right) di\right] \\
&\approx \frac{\lambda}{k+1} \exp\left[-\int_{m}^{k} \frac{\lambda(1+\beta \log(i+1))}{i+1} di\right] \\
&= \frac{\lambda e^{C(\lambda,\beta,m)}}{k+1} \exp\left[-\lambda \log(k+1) - \frac{\lambda \beta}{2} \log^2(k+1)\right],
\end{aligned}$$

where $C(\lambda, \beta, m)$ is a constant of integration equal to $\lambda \log(m+1) + \lambda \beta \log^2(m+1)/2$. In the first line, we are free to substitute $A(k) \sim k+1$ in the prefactor because the logarithmic term is negligible for large k; however, we unable to take the same liberty with $A(k)$ in the product since it ends up in an exponent. Note $f(x) \sim g(x)$ is used to denote that f is asymptotically equal to g with respect to x. In the next-to-last line, we appeal to the first-order Taylor approximation of the logarithm $\log(1+x) \approx x$. The last line is obtained by evaluating two elementary integrals. Hence $P(k)$, being proportional to $n(k)$, asymptotically follows the log-normal distribution, which completes the proof.

Supplementary Tables

Table S1. APS journal collection citation data growing network representations at different time resolutions. Each row summarises a growing network representation of the APS data. The representations are ordered from highest to lowest level of time resolution. Maximal resolution means that each article is mapped to a unique time-step. Daily resolution means that articles published on the same day are mapped to the same time-step; monthly and yearly resolution are similarly defined. In these instances, it is meaningful to distinguish citations for which the citing and cited article map to different time-steps (inter-time-step citations) from citations for which the citing and cited article map to the same time-step (intra-time-step citations). Minimal resolution means that all articles are mapped to a single time-step.

Resolution	Time-steps	Inter-time-step Citations	Intra-time-step Citations
Maximal	347,038	3,063,726	0
Daily	4,680	3,048,894	14,832
Monthly	1,287	3,047,818	15,908
Yearly	110	2,913,795	149,931
Minimal	1	0	3,063,726

Table S2. Bi-epochally resolved growing network representations of APS journal collection citation data. Each row summarises a growing network representation of some subset of the APS data. In each bi-epochal representation, the n_1 articles in time interval T_1 with m_1 citations are mapped to time-step $t = 1$, and the $n_2 = 14,692$ articles in $T_2 = 2000$ to $t = 2$. A total of m_2 citations from the n_2 articles at $t = 2$ fall on the n_1 articles at $t = 1$. Note that bi-epochal is used to refer to a growing network representation of bibliographic data consisting of exactly two time-steps.

T_1	n_1	m_1	m_2
1990-99	113,542	609,522	123,264
1980-99	181,173	1,311,276	148,709
1970-99	226,982	1,783,703	157,101
1893-99	282,667	2,441,817	164,458

Supplementary Figures

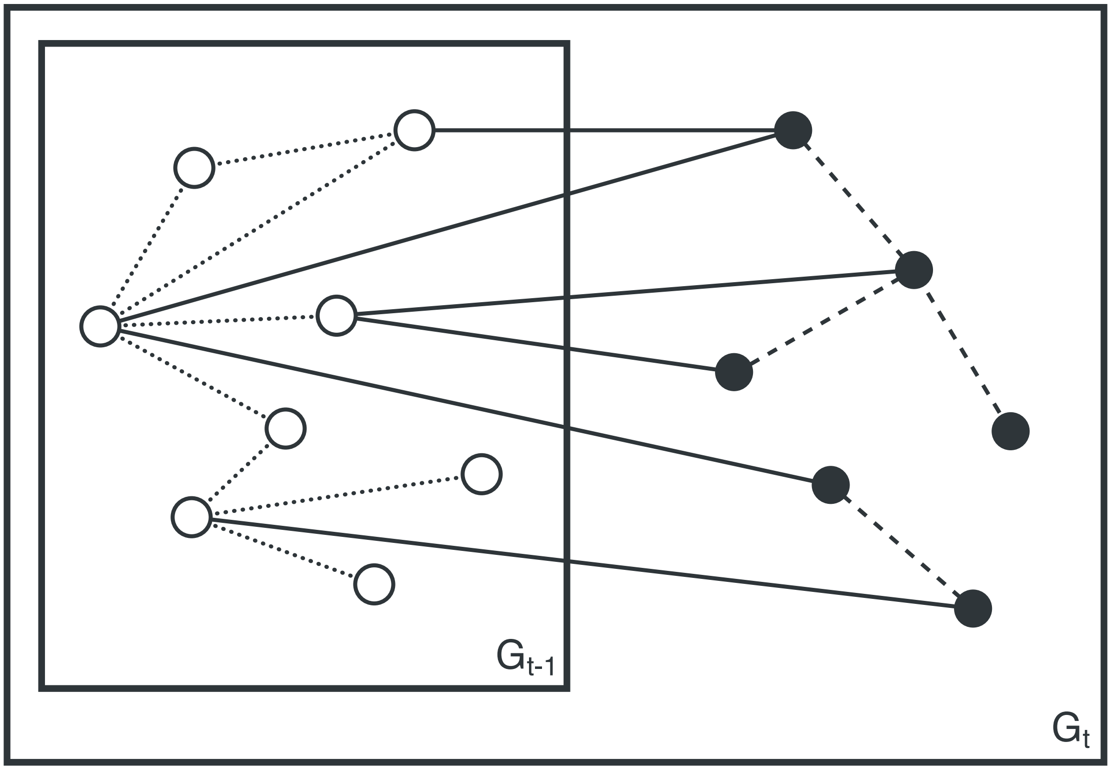

Figure S1. Schematic overview of a growing network at time-step t. The network G_t (outer rectangle) is generated by augmenting G_{t-1} (inner rectangle) with $n_t = 6$ nodes (closed circles) that have $m_t = 6$ edges (solid lines) connecting to the nodes of G_{t-1} (open circles), and $m_t' = 4$ edges (dashed lines) distributed among the newly added nodes. The edges internal to G_{t-1} are depicted as dotted lines.

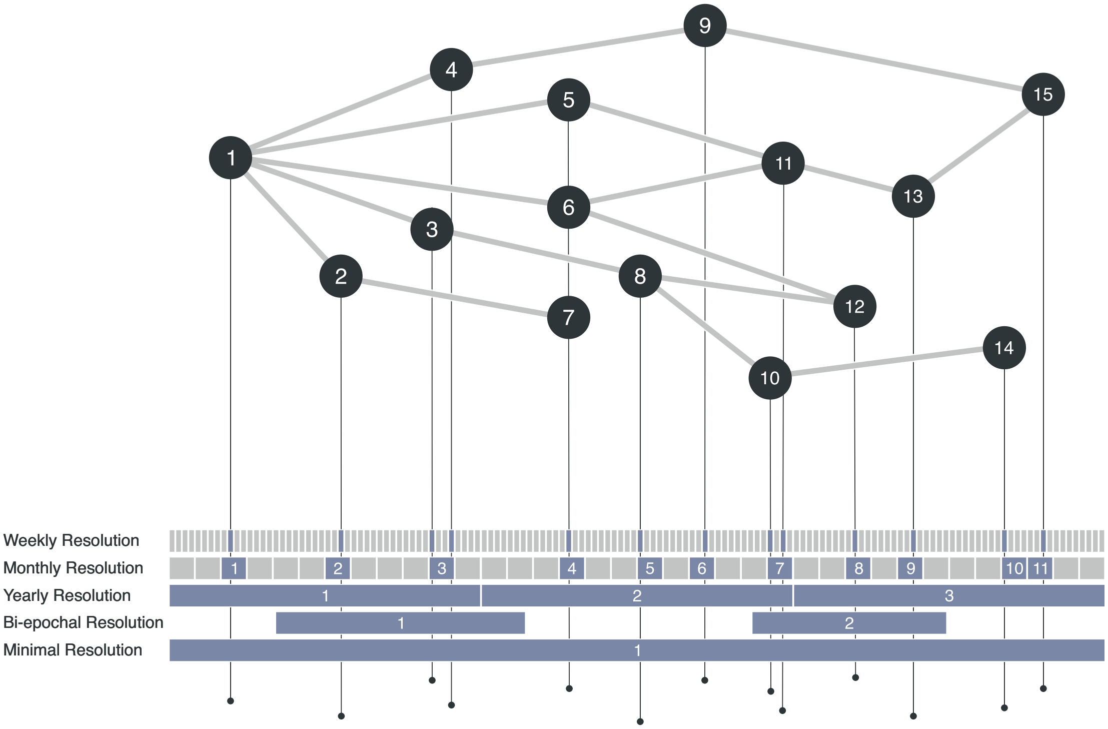

Figure S2. Schematic overview of time resolutions for an example network at differing levels of granularity. An example network consisting of 15 nodes (closed circles) and various edges (grey lines) is depicted above a number of possible time resolutions. A possible maximal resolution of the network is given by the numbering shown inside the individual nodes. Note that the nodes appearing at time-steps $t = 5, 6, 7$ occur at the same clock time. As such, the nodes in question can be mapped to $t = 5, 6, 7$ in any order and still technically yield a maximal resolution. The vertical lines show how the nodes map to various time resolutions of decreasing granularity. A depiction of the weekly resolution (top bar) of the network is shown in place of the daily resolution, of which we avail ourselves in the paper, for the sake of visual clarity. Each rectangle represents a calendar week; purple indicates that a node falls within the associated week, and grey, not. There are as many time-steps as purple rectangles (numbers not shown). Note that in this instance the three nodes occurring at the same clock time are all assigned to time-step $t = 5$. The monthly and yearly resolutions (second and third bar from the top, respectively) are most easily understood by analogy to the weekly resolution. In the bi-epochal resolution (second bar from bottom) example, two non-overlapping time intervals are selected in such a manner that only a sub-network of the original network is retained. Finally, in the minimal resolution (bottom bar) all the nodes of the network are mapped to a single time-step.

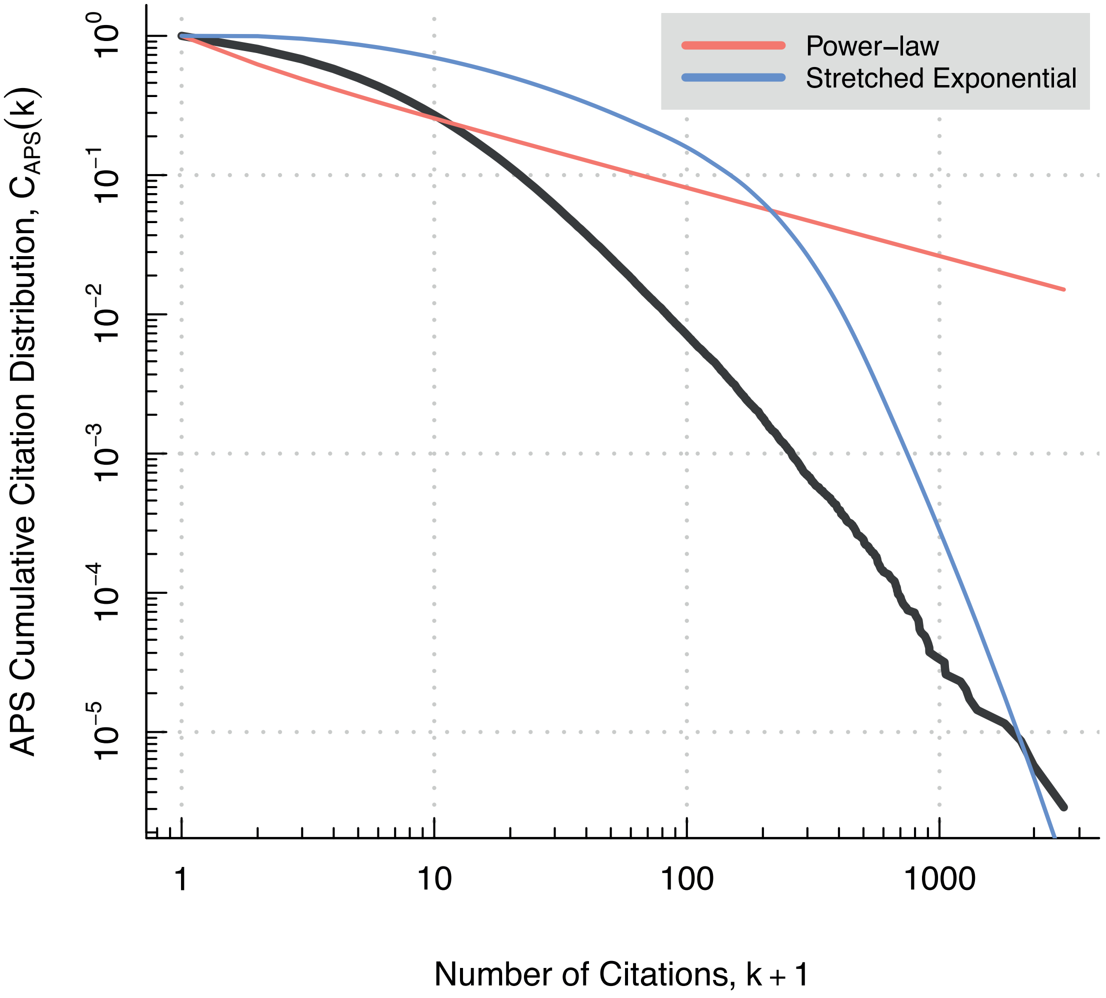

Figure S3. The APS cumulative citation distribution $C(k)$ for all publications dating from July 1893 up to and including June 2003. A power-law fit (red) is plotted alongside a stretched exponential fit (blue) predicted by Krapivsky's model when the attachment exponent α is taken to be 0.90. The value $\hat{\alpha} = 0.90$ was obtained by using Newman's method on the maximally resolved APS citation data.

A

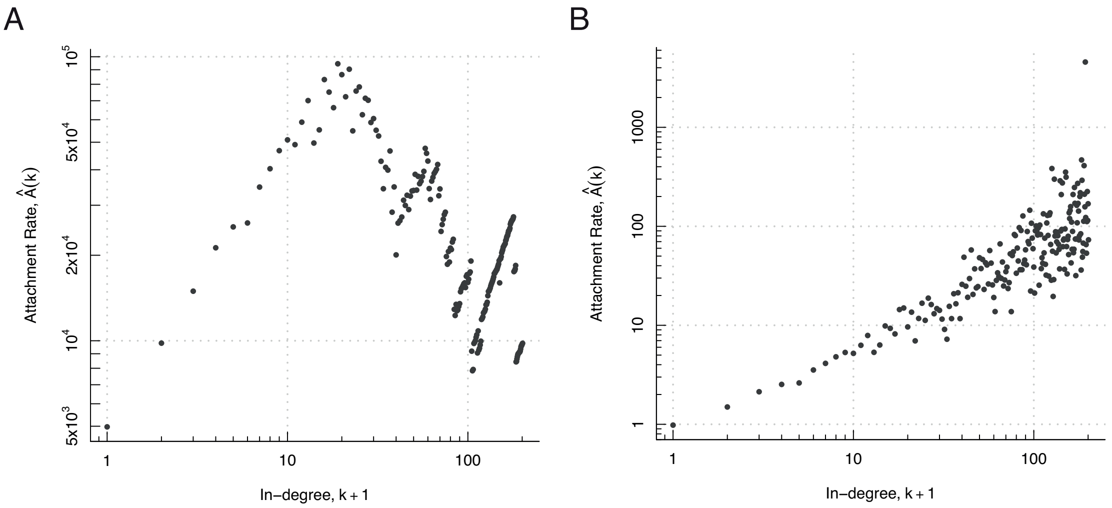

Figure S4. **(A) The uncorrected version of Newman's method**[4] is shown to introduce a waterfall effect in the tail region of k on a $10,000$ node Price's model network with $m = 1$. **(B) The corrected version of Newman's method**[5], as described in the main text, is shown to eliminate the waterfall effect when applied to the same Price's model network.

References

1. Newman, M. *Networks: An Introduction* (Oxford University Press, Inc., New York, NY, USA, 2010).

2. Krapivsky, P. L. & Redner, S. Organization of growing random networks. *Phys. Rev. E* **63**, 066123+ (2001). URL http://dx.doi.org/10.1103/physreve.63.066123. DOI 10.1103/physreve.63.066123.

3. Krapivsky, P. L., Redner, S. & Leyvraz, F. Connectivity of Growing Random Networks. *Phys. Rev. Lett.* **85**, 4629–4632 (2000). URL http://dx.doi.org/10.1103/physrevlett.85.4629. DOI 10.1103/physrevlett.85.4629.

4. Newman, M. E. J. Clustering and preferential attachment in growing networks. *Phys. Rev. E* **64** (2001). URL http://arxiv.org/abs/cond-mat/0104209. cond-mat/0104209.

5. Pham, T., Sheridan, P. & Shimodaira, H. Pafit: A statistical method for measuring preferential attachment in temporal complex networks. *PLoS ONE* **10**, e0137796 (2015). URL http://dx.doi.org/10.1371%2Fjournal.pone.0137796. DOI 10.1371/journal.pone.0137796.
